# Solvolytic recycling of unsaturated polyester resin-based sheet moulding composites

**DOI:** 10.12688/openreseurope.19012.1

**Published:** 2025-02-07

**Authors:** John van de Moosdijk, Annemieke van de Runstraat, Richard van Someren, Mark Roelands

**Affiliations:** 1 TNO, Sustainable Processes and Energy Systems, Kesslerpark 1, Rijswijk, 2288 GS, The Netherlands

**Keywords:** Solvolysis, composite, SMC, sheet moulding compounds, UPR, Unsaturated polyester resin, HSP, glass fiber

## Abstract

**Background:**

New regulations on low emission vehicles has incentivized a push towards reducing the weight of vehicles. While the implementation of lightweight Sheet Moulding Compounds (SMC’s) in the automotive industry is taking shape, a recycling strategy that does not downgrade the fibers is not commercially applied yet. This paper investigates a broad scope of reaction conditions for the solvolysis of SMC’s based on unsaturated polyester resins (UPR).

**Methods:**

The Hansen Solubily Parameter theory was used to model and select prospective solvents for the project. A method is disclosed for recovering the glass fibers from SMC’s, using base chemicals such as monoethoxyamine (MEA) and potassium hydroxide (KOH), and relatively mild conditions. Tensile testing is used to assess the effect of solvolysis on the fibers. Thermogravimetric analysis was used to determine residual material on the fibers.

**Results:**

The best solvolysis results were obtained with MEA/KOH at 170 °C. As a result of the mild conditions used, the strength of the fibers is not affected. TGA analysis shows that the removal of fiber sizing depends on the nature of the used catalyst. It also showed that the use of acetophenone as solvent raised the decomposition temperature of the resin

**Conclusions:**

An effective and mild method for the solvolysis of UPR based sheet moulding compounds was developed. The removal of the sizing of the fibers can be influenced by choosing an appropriate catalyst. It is postulated that acetophenone reacts with the resin and as a result makes it more thermally stable.

## Plain language summary

To reduce the weight of new cars and other vehicles, resin composites are used more and more instead of metal sheet parts. However, recycling these composites is still difficult, and they often end up in landfill or are incinerated. The research in this paper shows that it is possible to recycle them by reacting them with a mixture of monoethylamine and potassium hydroxide. As a result, the resin falls apart, and the glass fibers inside can be recovered without losing any strength.

## Introduction

Incentivized by European legislation on lower emissions from vehicles, there is a continuous push within the automotive industry towards light weight solutions. Sheet moulded compounds (SMC’s) are known to offer a significant reduction in weight compared to steel and aluminium parts with the same strength. They consist of a thermoset resin, a reinforcing fiber material, and optionally a filler. The material assessed in this publication consists of Unsaturated Polyester Resin (UPR), glass fiber reinforcement, and Al(OH) _3_ as a filler.

European legislation also requires automotive waste to be recycled instead of landfilled or incinerated. However, the existing recycling solutions for SMC’s often have limitations: e.g. incineration offers poor energy efficiency, generates polluting emissions, and reduced fiber strength, while mechanical recycling recovers only low value materials ( Naqvi *et al.*, 2018). Ideally, fibres are recovered with a low amount of residual resin, have close to virgin tensile properties, and good length retainment. If the fibers are to be reused in the same application, the retention of sizing on the fibers may be preferred, whereas it is preferably removed if the recycled fibers are destined for general use.

Solvolysis is a method for the recycling of polymers that involves breaking apart the polymer into oligomers or monomers. While solvolysis is already a fairly mature technology for thermoplastic polycondensates, unsaturated polyester resins are less well investigated ( Payne & Jones, 2021). The published research typically involves harsh conditions such as (near)-supercritical solvents, toxic reagents, or focuses on finely milled resin without regard for possible fiber recovery ( Chan *et al.*, 2019; Henry *et al.*, 2016; Wang *et al.*, 2021). The more widely reported solvolysis of anhydride crosslinked epoxy resins (ACER) can act as a source of inspiration for the solvolysis of UPR, since both involve breaking an ester bond. Similar to UPR, most earlier examples employ (near)-supercritical conditions, or finely milled materials. ( Kim *et al.*, 2017; Morin *et al.*, 2012; Tesoro & Wu, 1993). One of the first mentions of using larger pieces of ACER under relatively mild conditions still requires a temperature of 245 °C to achieve decomposition, both in the case of diethylene glycol/Ti(nBu) _4_ and monoethanolamine (MEA) ( El Gersifi *et al.*, 2006). Later, further investigation of solvent and matching of the Hansen Solubility Parameters (HSP) thereof to the epoxy resin resulted in improved yield and reduced reaction times, and Zhao *et al.* identify MEA as a preferred reagent ( Kuang *et al.*, 2018a; Kuang *et al.*, 2018b; Zhao *et al.*, 2022). Of special interest is the publication by Mu *et al.* that describes the use of a mixture of solvents to optimize the match in HSP between solvent and polymer ( Mu *et al.*, 2022). Finally, the role of the catalyst is also extensively researched, with mentions of both Lewis and Brønsted bases, acids, and metal complexes ( Wang *et al.*, 2015; Zhang *et al.*, 2022; Zhao *et al.*, 2021). The described methods involve milder conditions and reagents, and are more suitable to allow for the recovery of fibers.

**Figure 1.**
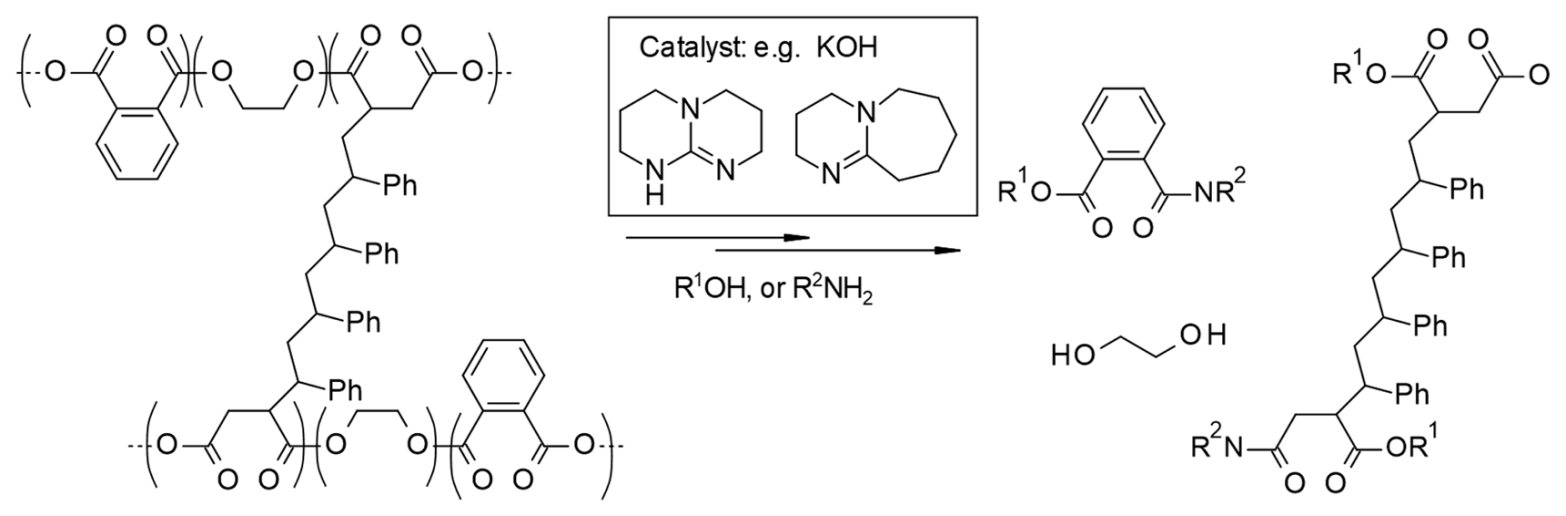
Solvolysis mechanism of UPR.

In this work, the suitability of MEA for the solvolysis of UPR is explored, and the effect of various reaction conditions on the yield is determined. A scoping of possible synergistic effects of solvent mixtures is presented in the data repository as Dataset 3. Additionally, the mechanical properties of recovered fibers is determined and compared to virgin material. A graphic representation of the proposed solvolysis of UPR is presented in Figure 1.

## Methods

All solvents were reagent grade solvents obtained from Merck. KOH, triazabicyclodecene (TBD), and 1,8-Diazabicyclo(5.4.0)undec-7-ene (DBU) were also purchased at Merck, and of at least 99% purity. Sheet moulding compound were provided by Batz, and consists of unsaturated polyester resin, E-glass fiber reinforcement, and Al(OH) _3_ as a filler.

The mixtures were prepared in 20 ml microwave vials, using cut SMC samples with dimensions of 2 × 1 cm and a thickness of 7 mm. The samples were heated at 170°C for 2h. After cooling down, the reaction mixtures were filtered on a Buchi filter and washed with 10ml methanol, 10ml water and then 2 more times with 10ml methanol. After drying them overnight in a vacuum oven, the weight of the solids were determined.

Larger scale testing was performed in a 1 liter glass reactor, using SMC samples of 5 × 10 cm. The reaction mixture was then left at 170 °C for 4 hours, to ensure full liberation of fibers. A selection of fibers was then submitted to tensile testing.

TGA was determined on a TA instruments TGA 55 machine, with a linear temperature ramp of 20 °C min ^-1^ from room temperature to 900 °C and under a N _2_ atmosphere. All raw TGA data is included in the data repository as Dataset 1.

Tensile testing was performed in tenfold on a Instron 5966 with a 100 N force cell, and a strain of 1 mm min ^-1^. The measurements were performed according to ASTM C1557. ( ASTM, n.d.). All raw tensile test data is included in the data repository as Dataset 2.

## Results

Initial scoping reactions with mixtures of solvents and MEA were performed to determine a starting point for further parameter optimization. It became clear that neat MEA provided the best results, as shown in Figure 2.2. In fact, no other solvent resulted in any significant weight reduction, although the experiments with acetophenone (ACP, Figure 2.3) did result in some disintegration of the composite sample. A full description of these scoping experiments is provided in the Supporting Information.

**Figure 2.**
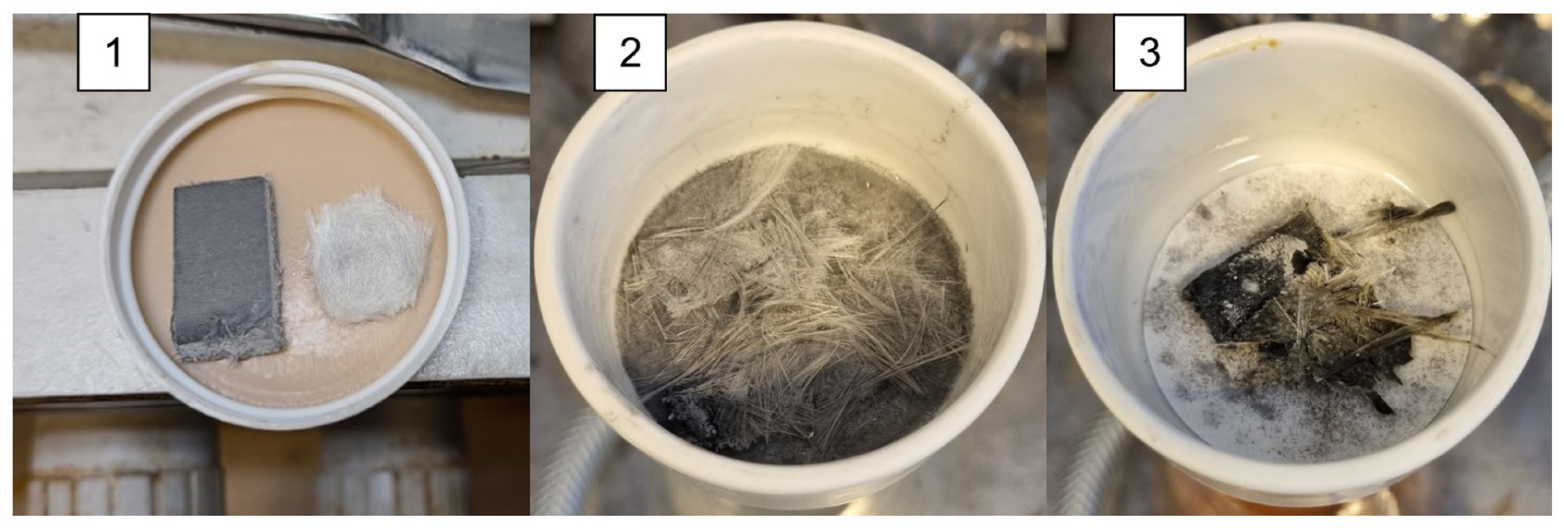
Untreated sample of SMC composite and the glass fibers inside ( **1**). Results of solvolysis in MEA/KOH ( **2**) and MEA/KOH/ACP 0.1/0.9 ( **3**).

Based on these initial results, a further scoping was performed regarding the influence of catalyst, temperature, reaction time and catalyst concentration. Since the HSP of a mix of solvents is simply the average of the components, a MEA/ACP mixture was tested in different ratio’s to see if the promising results could be further improved. An overview is presented in Table 1.

**Table 1.** Scoping of reaction conditions of solvolysis. ^1^ °C. ^2^ minutes. ^3^ mol L ^-1^. ^4^ Control experiment, same conditions as entries 3 and 4. Weights are reported in gram.

Entry	Temperature ^1^	MEA/ACP	Time ^2^	Conc. Cat. ^3^	wt. Before	wt. After	wt. change
1	170	1.0/0.0	120	0.35, DBU	1.166	1.119	-4%
2	170	1.0/0.0	120	0.35, TBD	1.143	0.947	-17%
3	170	1.0/0.0	120	0.35	0.967	0.695	-28%
4	170	1.0/0.0	120	0.35	1.082	0.858	-21%
5	170	1.0/0.0	120	0.2	1.072	0.875	-18%
6	170	1.0/0.0	120	0.1	1.145	1.002	-12%
7	160	1.0/0.0	120	0.35	1.074	0.907	-16%
8	150	1.0/0.0	120	0.35	0.941	0.826	-12%
9	170	0.7/0.3	120	0.35	0.968	0.961	-1%
10	170	0.5/0.5	120	0.35	1.066	1.084	2%
11	170	0.3/0.7	120	0.35	1.045	1.055	1%
12	170	1.0/0.0	30	0.35	0.972	0.939	-3%
13	170	1.0/0.0	60	0.35	1.024	0.818	-20%
14 ^4^	170	1.0/0.0	120	0.35	1.057	0.734	-31%

**Figure 3.**
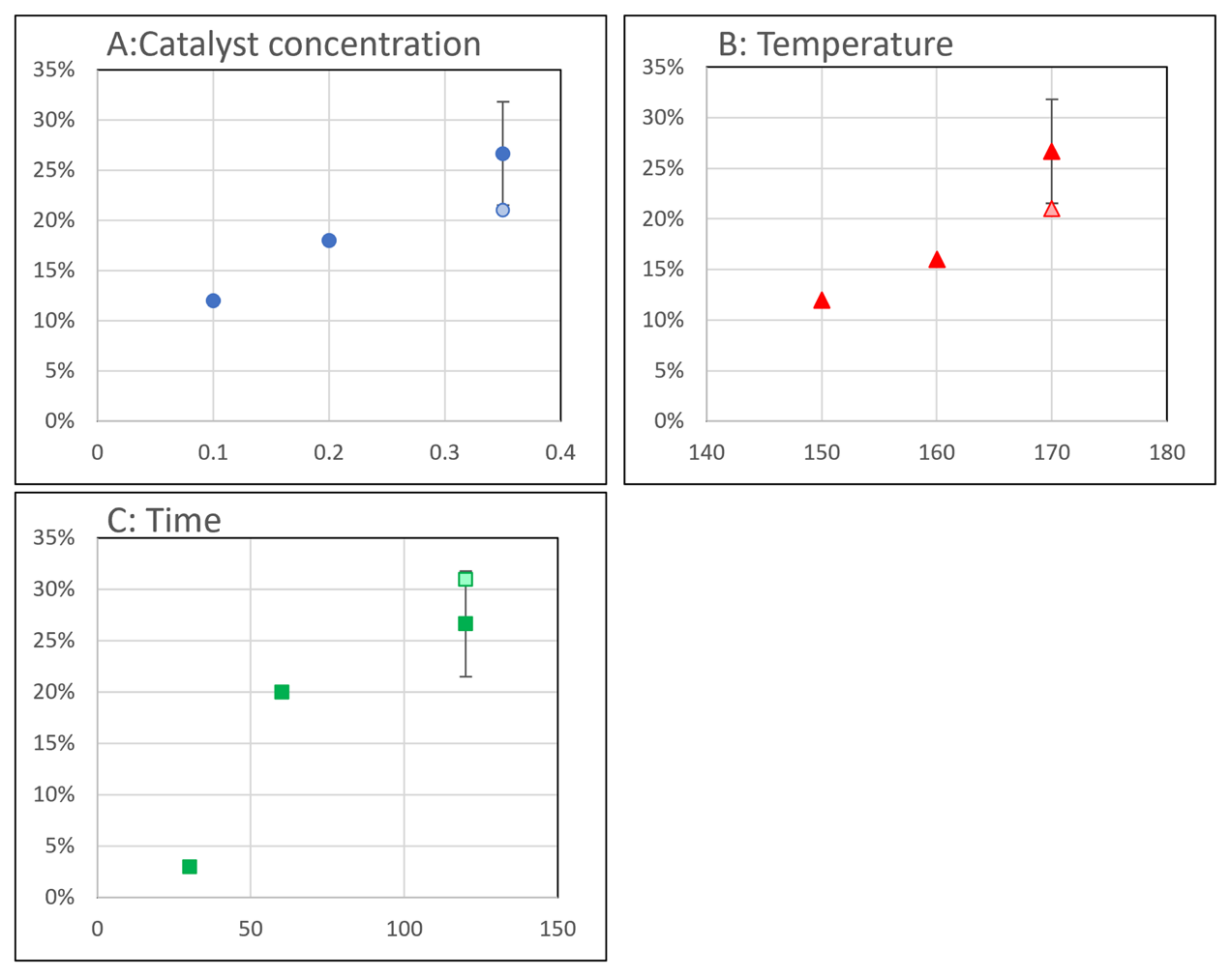
Graphs of the effects of catalyst concentration ( **A**), temperature ( **B**) and time ( **C**) on the weight reduction of the SMC after solvolysis. Light colored markers indicate the value of the experiment of the same run as the other two experiments.

The catalyst screening shows that KOH performs best with a weight reduction of the sample by 28 %, followed by TBD (17 %), and finally DBU with only 4 %. As seen in Figure 3, the rate of depolymerization increases with increasing catalyst concentration, time and temperature. It is interesting to note that there is a considerable spread on entries 3, 4 and 14, in which the (in)homogeneity of the samples is expected to play a role. As a result, precise conclusions on the kinetics of the solvolysis could not be made. Finally, different ratios of MEA/ACP as a solvent did not seem to offer any benefit to the reaction

Thermogravimetric analysis (TGA) and derivative thermogravimetry (DTG) were performed on the produced samples to assess the amount of residual resin on the fibers. The DTG curve of the SMC shows two distinct peaks, in which the peak with a maximum around 285 °C can be attributed to calcination of the filler Al(OH) _3_ to γ- Al _2_O _3_ with the release of water ( Du *et al.*, 2009), and the peak with a maximum of approximately 385 °C is attributed to the UPR matrix ( de Souza *et al.*, 2020; Hossain *et al.*, 2017). Mass loss below 230 °C is attributed to dehydration events.

**Figure 4.**
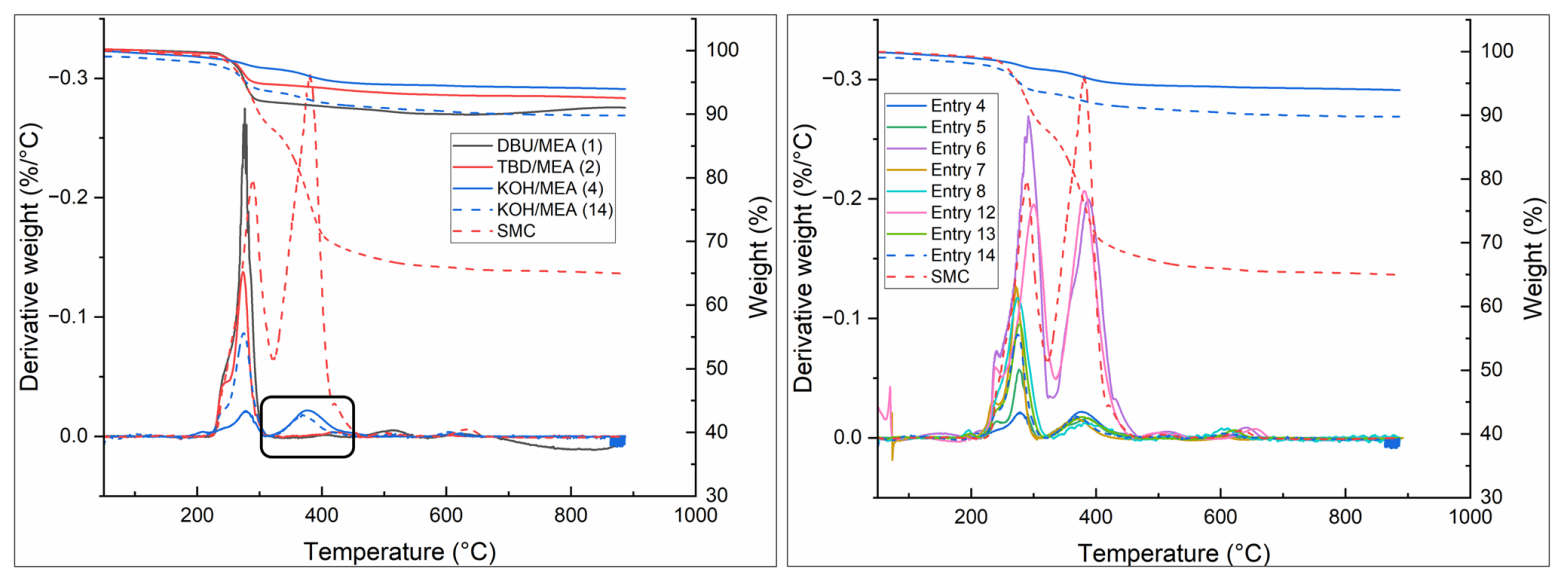
TGA and DTG analysis of solvolysis SMC samples. Left shows the comparison of the testes catalysts. Right shows a comparison of all KOH/MEA samples.

As shown in Figure 4 (black box), the fibers treated with TBD and DBU show a more complete removal of the resin compared to KOH, as indicated by the complete lack of signal at 385 °C. This is in contrast to the poorer results in bulk solvolysis. All samples that were treated with KOH as catalyst showed a similar amount of remaining resin on the fibres of 2 – 3 wt.%, as determined by the observed weight reduction in TGA and the integral of the DTG peak. It is likely that this is due to the sizing still being present on the fiber, even after solvolysis, while with TBD and DBU this is fully removed. Regarding the other variables, for all entries the intensity of the peak at 385 °C show either the same residual 2 – 3 wt. % as entries 3 and 14, or they are similar to the untreated material. These results suggest that the weight loss of samples can be fully attributed to the solvolysis of the bulk resin, and that fibres that are released do not need further reaction time to remove residual resin.

**Figure 5.**
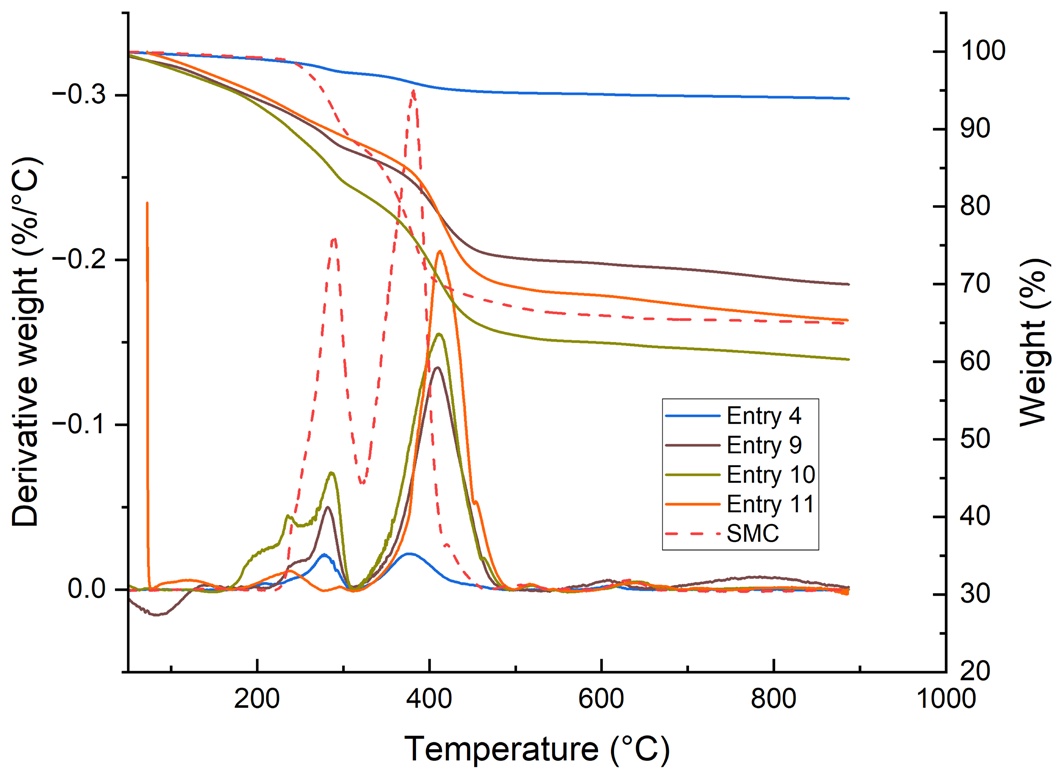
Effect of acetophenone on the degradation temperature of UPR.

Finally, the DTG graphs of the samples treated with MEA/ACP are presented in Figure 5. They show a shift of the peak temperature of the resin degradation event, with a similar peak intensity. Although some reaction of free deprotonated alcohol groups of the resin with ACP might be expected, this would only provide an endcap to the polymer chain. It was therefore surprising to see the distinct shift in degradation temperature in TGA. A publication by Yun *et al.* mentions the acetophenone assisted photocrosslinking of poly(ethylene terephthalate) under UV irradiation, which provides a possible explanation for the observed effect ( Yun & Jang, 2014). 

### Large scale reaction

**Figure 6.**
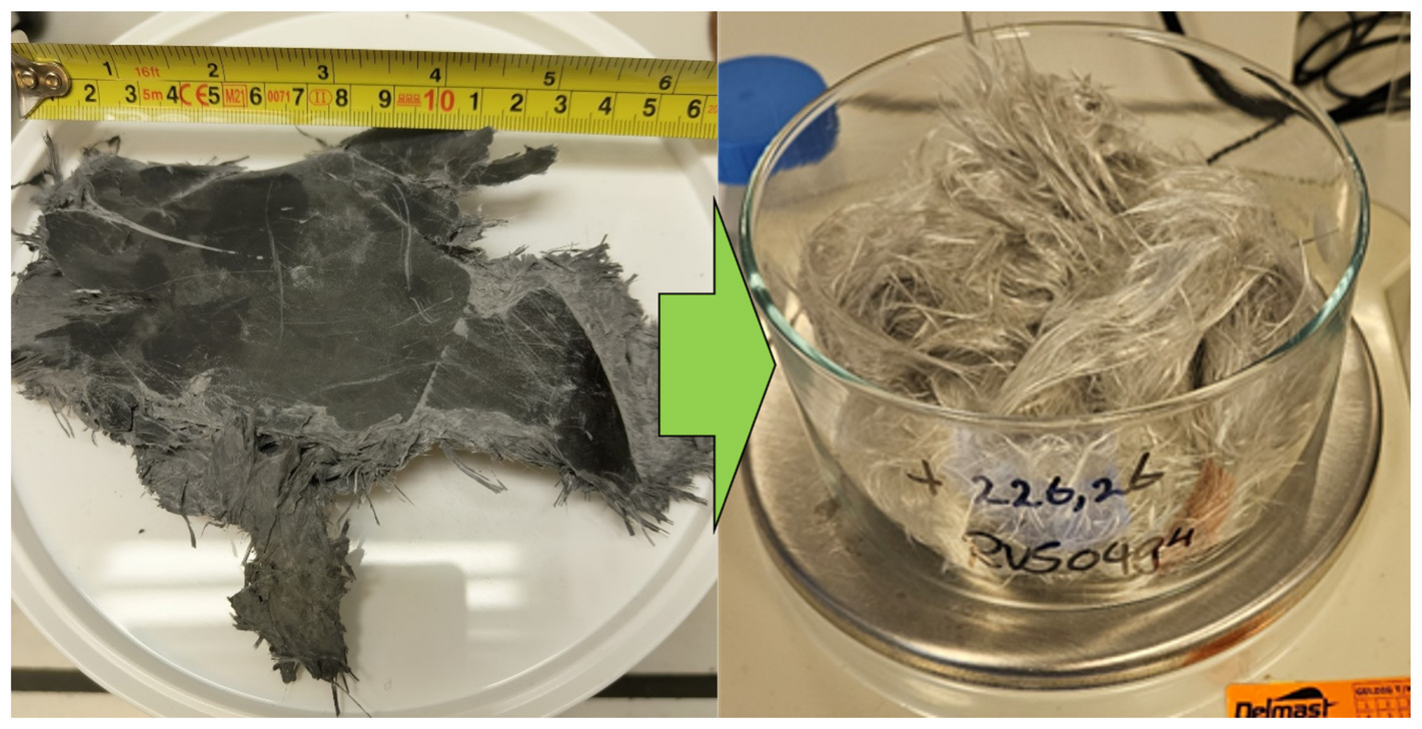
Large scale sample of SMC before and after solvolysis.

A larger scale experiment has been performed on a 5 × 10 cm SMC piece, using the optimized reaction conditions obtained from the scoping, i.e. 170 °C and 0.35 M KOH in neat MEA. As shown in Figure 6, effective decomposition of the resin was achieved. Since the aim of this experiment is to obtain clean fibers suitable for tensile testing, the reaction time was longer (4 hours), and the material was thoroughly washed. Clean fibers with a length of 5 cm were obtained, and submitted for tensile testing. The measured Young’s modulus of 80.2 ± 12.0 GPa and tensile strength of 2147 ± 350 MPa are close to the reported values for E type glass fibers, which are 70 – 80 GPa and 2000 – 3400 MPa respectively ( AZoM, n.d.; Hartman *et al.*, 1996). The raw data of the tensile tests can be found in the data repository Although no virgin fiber of sufficient length were available for a direct comparison, this indicates that the solvolysis did not induce significant deterioration of the mechanical properties of the recovered fibres. As a result, these fibers might be re-used in high-value applications such as in automotive and aerospace industries.

## Conclusion

A scoping of solvochemical recycling options for SMC’s based on unsaturated polyester and glass fibers was performed. Solvolysis under relatively mild conditions in the presence of a basic catalyst showed the technical feasiblity of the solvolysis process. While the use of the organic superbases DBU and TBD showed complete removal of the resin from the fiber, KOH gave better results in rapid bulk solvolysis while likely retaining the fiber sizing. This is desirable if the fibers are to be reused in high-end applications. Fibers recovered from the solvolysis process showed excellent properties with regard to tensile strength, similar to virgin strength.

## Ethics and consent

Ethical approval and consent were not required

## Data Availability

OSF Repository: Solvolytic recycling of Unsaturated Polyester Resin-based Sheet Moulding Composites. https://doi.org/10.17605/OSF.IO/TZPYH. ( van de Moosdijk, 2024) This project contains the following underlying data: Dataset 1. TGA data (underlying data) Dataset 2. Tensile test data (underlying data) Dataset 3. Supporting information on solvent scoping experiments (supporting data) Dataset 4. Figures used in this article Dataset 5. Tables used in this article Data are available under the terms of the Creative Commons Zero ""No rights reserved"" data waiver. ( Creative Commons, n.d.)

## References

[ref-1] ASTM: Standard Test Method for Tensile Strength and Young's Modulus of Fibers. *c1557-20*. (n.d.); Retrieved December 13, 2024. Reference Source

[ref-2] AZoM: E-glass. (n.d.); Retrieved May 1, 2024. Reference Source

[ref-3] Chan CH Wakisaka M Nishida H : Specific oligomer recovery behavior from cured unsaturated polyester by superheated steam degradation. *Polym Degrad Stab.* 2019;161:1–6. 10.1016/j.polymdegradstab.2018.12.025

[ref-4] Creative Commons: No Title. (n.d.); Retrieved December 15, 2024. Reference Source

[ref-5] de Souza LGM da Silva EJ Meira de Souza LGV : Obtaining and characterizing a polyester resin and cement powder composites. *Mat Res.* 2020;23(5). 10.1590/1980-5373-MR-2018-0894

[ref-6] Du X Wang Y Su X : Influences of pH value on the microstructure and phase transformation of aluminum hydroxide. *Powder Technol.* 2009;192(1):40–46. 10.1016/j.powtec.2008.11.008

[ref-7] El Gersifi K Durand G Tersac G : Solvolysis of bisphenol A diglycidyl ether/anhydride model networks. *Polym Degrad Stab.* 2006;91(4):690–702. 10.1016/j.polymdegradstab.2005.05.021

[ref-8] Hartman D Greenwood ME Miller DM : High strength glass fibers. *Agy.* 1996;1–11.

[ref-9] Henry L Schneller A Doerfler J : Semi-continuous flow recycling method for carbon fibre reinforced thermoset polymers by near- and supercritical solvolysis. *Polym Degrad Stab.* 2016;133:264–274. 10.1016/j.polymdegradstab.2016.09.002

[ref-10] Hossain MM Elahi AHMF Afrin S : Thermal aging of unsaturated polyester composite reinforced with e-glass nonwoven mat. *Autex Research Journal.* 2017;17(4):313–318. 10.1515/aut-2016-0007

[ref-11] Kim KW Lee HM An JH : Recycling and characterization of carbon fibers from carbon fiber reinforced epoxy matrix composites by a novel super-heated-steam method. *J Environ Manage.* 2017;203(Pt 3):872–879. 10.1016/j.jenvman.2017.05.015 28506669

[ref-12] Kuang X Shi Q Zhou Y : Dissolution of epoxy thermosets: *via* mild alcoholysis: the mechanism and kinetics study. *RSC Adv.* 2018a;8(3):1493–1502. 10.1039/c7ra12787a 35540886 PMC9077052

[ref-13] Kuang X Zhou Y Shi Q : Recycling of epoxy thermoset and composites via good solvent assisted and small molecules participated exchange reactions. *ACS Sustain Chem Eng.* 2018b;6(7):9189–9197. 10.1021/acssuschemeng.8b01538

[ref-14] Morin C Loppinet-Serani A Cansell F : Near- and supercritical solvolysis of Carbon Fibre Reinforced Polymers (CFRPs) for recycling carbon fibers as a valuable resource: state of the art. *J Supercrit Fluids.* 2012;66:232–240. 10.1016/j.supflu.2012.02.001

[ref-15] Mu Q An L Hu Z : Fast and sustainable recycling of epoxy and composites using mixed solvents. *Polym Degrad Stab.* 2022;199: 109895. 10.1016/j.polymdegradstab.2022.109895

[ref-16] Naqvi SR Prabhakara HM Bramer EA : A critical review on recycling of end-of-life carbon fibre/glass fibre reinforced composites waste using pyrolysis towards a circular economy. *Resour Conserv Recycl.* 2018;136:118–129. 10.1016/j.resconrec.2018.04.013

[ref-17] Payne J Jones MD : The chemical recycling of polyesters for a circular plastics economy: challenges and emerging opportunities. *ChemSusChem.* 2021;14(19):4041–4070. 10.1002/cssc.202100400 33826253 PMC8518041

[ref-18] Tesoro G Wu Y : Chemical products from cured unsaturated polyesters. *Advances in Polymer Technology.* 1993;12(2):185–196. 10.1002/adv.1993.060120206

[ref-19] van de Moosdijk JHW : Solvolytic recycling of Unsaturated Polyester Resin-based Sheet Moulding Composites. [Dataset].2024. 10.17605/OSF.IO/TZPYH PMC11880758 40046797

[ref-20] Wang B Wang X Xu N : Recycling of carbon fibers from unsaturated polyester composites via a hydrolysis-oxidation synergistic catalytic strategy. *Compos Sci Technol.* 2021;203: 108589. 10.1016/j.compscitech.2020.108589

[ref-21] Wang Y Cui X Ge H : Chemical Recycling of Carbon Fiber Reinforced Epoxy Resin Composites via Selective Cleavage of the Carbon-Nitrogen Bond. *ACS Sustain Chem Eng.* 2015;3(12):3332–3337. 10.1021/acssuschemeng.5b00949

[ref-22] Yun DW Jang J : Acetophenone-assisted main-chain photocrosslinking of poly(ethylene terephthalate). *J Appl Polym Sci.* 2014;131(2): 39802. 10.1002/app.39802

[ref-23] Zhang N Wu S Wang C : Efficient catalytic degradation of anhydride-cured epoxy resin by amphiphilic molecule catalysts. *Green Chemistry.* 2022;24(19):7395–7402. 10.1039/D2GC02248F

[ref-24] Zhao Q An L Li C : Environment-friendly recycling of CFRP composites via gentle solvent system at atmospheric pressure. *Compos Sci Technol.* 2022;224:109461. 10.1016/j.compscitech.2022.109461

[ref-25] Zhao W An L Wang S : Recyclable high-performance epoxy-anhydride resins with DMP-30 as the catalyst of transesterification reactions. *Polymers.* 2021;13(2):296. 10.3390/polym13020296 33477708 PMC7831910

