# Supplementary material for: Solvolytic recycling of unsaturated polyester resin-based sheet moulding composites
Source: Open Res Eur. 2025 Feb 7;5:44. [Version 1] doi: 10.12688/openreseurope.19012.1 (PMC11880758; doi:10.12688/openreseurope.19012.1)
Supplement: Peer review history

# Reviewer report: Reviewer response for version 1

**DOI:** 10.21956/openreseurope.20573.r51133

**Published:** 2025-04-15

**Authors:** Andrey E. Krauklis

**Affiliations:** 1 University of Latvia, Jelgavas street, Latvia

This study by van de Moosdijk et al. addresses an important challenge in the recycling of unsaturated polyester resin (UPR)-based sheet moulding compounds (SMCs), which are increasingly used in automotive applications due to their lightweight nature. Despite their advantages, these composites pose recycling difficulties, especially in preserving the strength and usability of the embedded glass fibers. The authors propose a solvolytic recycling method using monoethanolamine (MEA) and potassium hydroxide (KOH) under relatively mild conditions (170 °C), aiming to recover intact, high-performance fibers suitable for reuse.

 The work is clearly and logically presented, with a solid foundation in current literature. The authors provide a comprehensive overview of previous work on solvolysis in both polyester and epoxy resins, helping to position their contribution within the broader research landscape. Their use of Hansen Solubility Parameters (HSP) to guide solvent selection is a noteworthy strength, as is their systematic evaluation of solvent mixtures, catalyst types, concentrations, and processing times. MEA/KOH emerged as the optimal combination, effectively depolymerizing the matrix while preserving fiber strength, as demonstrated through thermogravimetric analysis (TGA) and tensile testing. The recovered fibers showed tensile strength and modulus values close to those of virgin E-glass, suggesting minimal degradation during processing.

 However, while the methodology is largely reproducible and well-documented, some details warrant clarification. For instance, variability in solvolysis outcomes across seemingly identical conditions is noted but not deeply analyzed. Figures presenting experimental data, such as Figure 3, only include error bars in isolated instances, making it difficult to fully assess experimental consistency. Moreover, the assumption that the tested fibers are equivalent to standard E-glass is not explicitly stated, and recent references for glass fiber strength could strengthen the case.

 The conclusions are generally well-supported, but the authors slightly overextend in suggesting high-end reuse without testing interfacial compatibility or performance in new composites. Additionally, while the raw data is available through an OSF repository, clearer alignment between specific data points and corresponding figures in the manuscript would enhance reproducibility. The environmental and economic trade-offs of solvent use and recovery are not addressed, which would be essential for assessing the real-world feasibility of scaling this approach.

 In summary, this is a valuable contribution to the field of composite recycling, offering a milder and more fiber-friendly route for recovering UPR-based materials. Addressing minor methodological clarity and tempering some of the reuse claims would strengthen the work. Inclusion of further environmental impact analysis and more detailed error treatment in data presentation would also enhance its scientific rigor and practical relevance.

Is the study design appropriate and does the work have academic merit?

Yes

Is the work clearly and accurately presented and does it cite the current literature?

Yes

If applicable, is the statistical analysis and its interpretation appropriate?

Yes

Are all the source data underlying the results available to ensure full reproducibility?

Partly

Are the conclusions drawn adequately supported by the results?

Partly

Are sufficient details of methods and analysis provided to allow replication by others?

Partly

Reviewer Expertise:

Materials Science, Polymers, Composites, Modelling

I confirm that I have read this submission and believe that I have an appropriate level of expertise to confirm that it is of an acceptable scientific standard.

# Reviewer report: Reviewer response for version 1

**DOI:** 10.21956/openreseurope.20573.r51139

**Published:** 2025-03-04

**Authors:** Farshid Nazemi

**Affiliations:** 1 School for Engineering Mater, Transport, and Energy, Arizona State University, Arizona, Arizona, USA

This paper introduces a novel recycling technique for recovering unsaturated polyester resin-based sheet molding composites. The experimental work is thoroughly examined, and the results substantiate the authors’ claims. However, I have two major concerns:

1. The authors assert that the quality of the recovered fibers is equivalent to that of virgin fibers. However, have they tested the material for subsequent cycles of sheet molding composite production? How many times can the fibers be successfully recovered using the proposed technique before degradation occurs? Is the limit three, five, ten, or theoretically infinite cycles? This aspect is critical in assessing the long-term viability and true benefit of the recycling process.
2. The study would greatly benefit from a preliminary life cycle analysis to demonstrate the advantages of solvolytic recycling over landfilling throughout the life cycle of SMCs. Additionally, given the use of a solvent, what are the associated environmental and economic challenges? How is the solvent recovered post-processing? Ultimately, does this approach offer a genuine improvement over the linear economy (i.e., landfilling)? In some cases, poorly executed circular strategies can inadvertently lead to greater environmental burdens rather than mitigating them.

Is the study design appropriate and does the work have academic merit?

Yes

Is the work clearly and accurately presented and does it cite the current literature?

Yes

If applicable, is the statistical analysis and its interpretation appropriate?

Yes

Are all the source data underlying the results available to ensure full reproducibility?

Yes

Are the conclusions drawn adequately supported by the results?

Partly

Are sufficient details of methods and analysis provided to allow replication by others?

Yes

Reviewer Expertise:

Sustainable Engineering, Life cycle assessment, techno-economic analysis, circular economy, process systems engineering

I confirm that I have read this submission and believe that I have an appropriate level of expertise to confirm that it is of an acceptable scientific standard, however I have significant reservations, as outlined above.

# Reviewer report: Reviewer response for version 1

**DOI:** 10.21956/openreseurope.20573.r51135

**Published:** 2025-03-04

**Authors:** Roksana Muzyka

**Affiliations:** 1 Silesian University of Technology, Gliwice, Poland

The article presents significant research findings concerning the solvolysis of unsaturated polyester resins. The authors clearly analyze the impact of various reaction parameters, such as process duration and the use of different catalytic compounds.

 One of the main strengths of this work is the optimization of the research, which ensures not only the reproducibility of the results but also facilitates process scaling. The information provided is valuable and constitutes an important contribution to the development of recycling technologies for composites based on unsaturated polyester resins.

 However, I have a few editorial comments. I recommend that the authors review the text to eliminate minor language errors, such as typos, missing punctuation marks, and incorrect use of capital letters within sentences. Additionally, it would be advisable to verify the accuracy of citations, especially concerning specific standards.

 In conclusion, the article makes a significant contribution to research on solvolytic recycling. After implementing the suggested linguistic and editorial corrections, the work is suitable for indexing.

Is the study design appropriate and does the work have academic merit?

Yes

Is the work clearly and accurately presented and does it cite the current literature?

Yes

If applicable, is the statistical analysis and its interpretation appropriate?

Not applicable

Are all the source data underlying the results available to ensure full reproducibility?

Partly

Are the conclusions drawn adequately supported by the results?

Yes

Are sufficient details of methods and analysis provided to allow replication by others?

No

Reviewer Expertise:

chemical recycling, solvolysis, oxidative liquefaction, pyrolysis, chromatography, carbon materials,

I confirm that I have read this submission and believe that I have an appropriate level of expertise to confirm that it is of an acceptable scientific standard, however I have significant reservations, as outlined above.

# Reviewer report: Reviewer response for version 1

**DOI:** 10.21956/openreseurope.20573.r51136

**Published:** 2025-02-25

**Authors:** Norshah Aizat Shuaib

**Affiliations:** 1 University Malaysia Perlis, Perlis, Malaysia

The paper addresses an important topic on the recovery of composite waste. With stricter legislation, especially in automotive applications, this study is particularly timely. While most recent studies focus on carbon fiber, this paper examines glass fiber, which is more abundant in volume than carbon fiber. The authors present interesting results on using chemicals to recover the fiber with minimal damage and residual resin. The methodology employed is robust, although the study would be more compelling if the recovered fiber were tested mechanically and explored for new applications. This could serve as a valuable direction for future investigation.

 Some comments for improvement of the script:

1. While other studies focus on carbon fiber due to its higher commercial value and price, the authors should explain why the consideration of glass fiber should not be overlooked. A comparison of waste volumes between the two fibers could help justify the importance of investigating glass fiber.
2. In the brief literature review, the authors should clarify the selection of solvents. On what basis were they chosen?
3. Safety precautions while handling chemicals should also be highlighted.
4. In Figure 3, why does only one point in each graph contain an error bar?
5. The authors should state their assumptions, specifically mentioning that it is assumed the type of glass fiber in the SMC is similar to what is reported in  ( AZoM, n.d.; Hartman *et al.*, 1996). Additionally, I suggest using more recent references, as the strength of glass fiber may have changed over the past 30 years.
6. It would be beneficial to mention in the conclusion that the study does not consider reuse applications, which could be explored in future research. Fiber strength alone is not a sufficient indicator of usability in new applications, as the recycling process might deteriorate the fiber sizing or surface, leading to poor interfacial bonding between the fiber and a new resin.

Is the study design appropriate and does the work have academic merit?

Yes

Is the work clearly and accurately presented and does it cite the current literature?

Partly

If applicable, is the statistical analysis and its interpretation appropriate?

Not applicable

Are all the source data underlying the results available to ensure full reproducibility?

Yes

Are the conclusions drawn adequately supported by the results?

Yes

Are sufficient details of methods and analysis provided to allow replication by others?

Yes

Reviewer Expertise:

composite recycling, green manufacturing, life cycle assessment

I confirm that I have read this submission and believe that I have an appropriate level of expertise to confirm that it is of an acceptable scientific standard.
